# Comparing clinical presentation, viremia, and immunological factors at various severity presentations in hospitalized children affected by COVID‐19: A cross‐sectional study

**DOI:** 10.1002/hsr2.1259

**Published:** 2023-05-09

**Authors:** Marzieh Jamalidoust, Seyedeh Sedigheh Hamzavi, Eslam Shorafa, Mandana Namayandeh, Laiba Batool, Seyedeh Narges Abootalebi

**Affiliations:** ^1^ Department of Virology, Professor Alborzi Clinical Microbiology Research Center, Namazi Hospital Shiraz University of Medical Sciences Shiraz Iran; ^2^ Professor Alborzi Clinical Microbiology Research Center Namazi Hospital, Shiraz University of Medical Sciences Shiraz Iran; ^3^ School of Medicine Shiraz University of Medical Sciences Shiraz Iran; ^4^ Pediatric Intensivist, Intensive Care Unit division, Department of Pediatrics, School of Medicine Shiraz University of Medical Sciences Shiraz Iran; ^5^ Biotechnology Research Center Shiraz University of Medical Sciences Shiraz Iran

**Keywords:** COVID‐19, paraclinical data, pediatrics, SARS‐CoV‐2, severity, viremia

## Abstract

**Background and Aims:**

Although SARS‐CoV‐2 infection usually leads to mild COVID‐19 in children, sometimes it causes serious complications, especially in those with underlying diseases. Several factors have been identified in determining disease severity in adults, and limited studies have been conducted in children. The prognostic implications of SARS‐CoV‐2 RNaemia as an important factor in determining disease severity in children are not well understood.

**Methods:**

In this study, we aimed to prospectively assess the relationship between disease severity and immunological factors and viremia in 47 COVID‐19 hospitalized children. In this research, 76.5% of children experienced mild and moderate COVID‐19, while 23.5% experienced severe and critical forms of the disease.

**Results:**

The presence of underlying diseases in different groups of pediatric patients differed significantly from each other. On the other hand, clinical symptoms such as vomiting and chest pain as well as laboratory parameters including erythrocyte sedimentation rate were significantly different in different groups of patients. Viremia was seen in only two children, and this had no significant relationship with the severity of COVID‐19.

**Conclusion:**

In conclusion, our data confirmed that COVID‐19 severity differed in SARS‐CoV‐2 infected children. Some clinical presentation and lab data parameters were different in various presentation of patients. Viremia was not associated with severity in our study.

## 1 INTRODUCTION

The new emerged SARS‐CoV‐2 infection, which has affected a wide range of adults and children worldwide, belongs to β‐coronaviruses and it is respiratory in nature with pulmonary effects although the clinical and disease course varies on a case‐by‐case basis.^1^, ^2^

Generally, children are the main population group affected by viral respiratory infections. However, the pediatric population has not been primarily affected by the pandemic, as was first noted during the first COVID‐19 pandemic wave.^3^

Children and adolescents who contract SARS‐CoV‐2 typically experience less severe illness and a lower mortality rate than adults. The most frequent clinical symptoms in newborns and infants are fever (53%–59%) and cough (48%–56%), while the less frequent symptoms are gastrointestinal, neurological, and upper and lower respiratory infection.^4^ However, most mortality from SARS‐CoV‐2 infection has been observed in adult and pediatric patients with underlying medical condition. The most important of these were diabetes, cardiovascular disease, hypertension, and chronic bronchitis.^5^

Unlike adults, where old age is an independent risk factor for severity and mortality, children at very young ages are considered a risk factor for severity, although this has recently been questioned, and MIS‐c is associated with older age.

Although pediatric cases of COVID‐19 have not revealed a clear pattern of laboratory findings related to disease severity, lymphopenia appears to be a risk factor for severe disease in children. Immunization history, vitamin D levels, RSV coinfection, and genetic polymorphisms are different factors that play a role in determining the severity of COVID‐19 in children.^6^ Information on other factors that influence disease severity, such as viral load in children, is limited.

Analysis of SARS‐CoV‐2 RNA in the nasopharynx, often incorrectly referred to as “viral load,” does not show a consistent association with asymptomatic versus symptomatic disease or symptomatic disease severity. In contrast, some studies have shown that there is a direct relationship between the presence of virus in the blood and disease severity in adults, but less information is available on the amount of virus in the blood in children and its relationship to the severity of the disease.^3^, ^7^, ^8^, ^9^, ^10^, ^11^, ^12^, ^13^

Comprehensive and detailed data on pediatric patients are limited or incomplete, even as the number of COVID‐19 cases continues to rise worldwide. SARS‐CoV‐2 infected children often develop Kawasaki‐like disease and multiorgan involvement.^14^

This study first aimed to evaluate the severity status and outcome of the disease and also to identify the most confounding factors in the morbidity and mortality of pediatric COVID‐19‐affected cases.

The most important factors used to determine the severity of the disease investigated were the paraclinical findings, viremic status, and the patients' underlying disease.

## 2 MATERIALS AND METHODS

### 2.1 Patients

In this cross‐sectional study, we prospectively followed up 47 pediatric patients from August 23, 2021 to November 21, 2021. The study was conducted at Nemazee Hospital, as a referral hospital which provides adult and pediatric tertiary care, affiliated to Shiraz University of Medical Sciences (SUMS), Shiraz, Iran. It was ethically approved by SUMS, Shiraz, Iran (IR.SUMS.REC.1400.691). Participants from 15 days to 17 years of age with confirmed SARS‐CoV‐2 infection were included in this study. All participants' legal guardians provided written informed consent and the study was conducted in accordance with the guidelines of the declaration of Helsinki.

The predefined primary endpoint was a composite of viremia and COVID‐19 severity. Participants were eligible for enrollment after meeting selection criteria, including: (1) Child (male or female) aged 1 day to 18 years, (2) a written consent to participate in the study, (3) a positive nasopharyngeal PCR test for COVID‐19, and (4) being hospitalized at different pediatric wards.

A confirmed case of COVID‐19 was defined as a positive SARS‐CoV‐2 real‐time reverse transcription‐polymerase chain reaction (rRT‐PCR) result using a nasopharyngeal swab.

Blood oxalate samples from patients with confirmed COVID‐19 who were admitted to the Pediatric Emergency Unit and Pediatric Intensive Care Unit were obtained and transferred to the Corona Lab at the Clinical Microbiology Research Center while maintaining the cold chain.

Two hundred microliters of each serum was extracted using the BehPrep viral nucleic acid extraction kit (AAAA15044000; Tehran, Iran). Nucleic acids extracted from each sample were amplified using rRT‐PCR from Pishtaz commercial kit (Pishtaz Teb PT. COVID.100; Tehran, Iran). This assay was performed using specific regions of the viral genome, RdRp (FAM‐tagged), N (HEX‐tagged), and internal control of RNaseP (ROX‐labeled) on an Applied Biosystems StepOne Plus real‐time PCR instrument (ABI, Thermo Fisher Scientific).^15^

Patients were divided into four groups based on SUMS guidelines that correspond to the World Health Organization (WHO) 10‐point ordinal scale: mild, moderate, severe, and critical, based on clinical findings, severity of pneumonia, respiratory failure, shock, and other organ failures.

Demographic characteristics, initial symptoms, clinical signs, laboratory findings, disease severity, and 60‐day mortality rates were recorded and followed by a general physician (Laiba Batool).

White blood cell count (WBC), lymphocyte, neutrophil and platelet count, erythrocyte sedimentation rate (ESR), d‐dimer, C‐reactive protein (CRP), lactate dehydrogenase (LDH), ferritin, troponin, procalcitonin, and hemoglobin levels were measured for the patients as the main immunological factors.

In this study, the majority of patients received Remdesivir and different types of antibiotics.

### 2.2 Statistical analysis

The Statistical Package for Social Sciences (SPSS version 18.0, SPSS Inc.) was used for statistical analysis. The normality of the data was assessed using Kolmogorov–Smirnov's test. Correlations between parametric and nonparametric data and severity in different groups were assessed using one‐way analysis of variance (ANOVA) and Kruskal–Wallis test, respectively. The results were presented in terms of mean ± Standard deviation (±SD).

## 3 RESULTS

To validate the primary objective of this study, the relationship between clinical severity and lab data as well as viremia was analyzed. Forty‐seven patients (25 males, 22 females) were followed for 2 months during the outbreak of the Delta virus strain in Iran. The patients included in the study ranged in age from 15 days to 17 years, with a mean of 9.78 years (SD ± 5.73). As shown in Table 1, the patients were classified into four categories based on clinical manifestations, ventilator use, oxygen with reservoir, vasoactive drug use, and IVIG or corticosteroid therapy.

**Table 1:** Demographic and clinical characteristics of SARS‐CoV‐2 infected children (*N* = 47).

Characteristics	Mild ill (*N* = 28)	Moderate ill (*N* = 8)	Severe ill (*N* = 8)	Critically ill (*N* = 3)	*p*‐Value
Age, SD (year)	9.7 (5.3)	9.3 (6.2)	9.7 (6.4)	8.7 (8.4)	0.47
Male/Female	15/13	5/3	3/5	2/1	0.75
Comorbid conditions, no. (%)	14 (50)	8 (100)	5 (62)	1 (33)	**0.02**
Hospital stay, mean (±SD)	13.8 (±17.9)	12.3 (±7.08)	8.3 (±5.8)	9	
60‐day mortality rate no. (%)	1 (3.4)	‐	1 (14.3)	‐	
Detectable SARS‐CoV‐2 viremia, no. (%)	‐	‐	2 (20)	‐	0.099
COVID‐19 treatment Remdesivir, n (%)	11 (38)	4 (50)	4 (57)	3 (100)	
**Symptoms**					
Fever, no. (%)	17 (61)	5 (62)	3 (37)	2 (67)	0.73
Cough, no. (%)	11 (39)	3 (37)	4 (50)	2 (67)	0.82
Shortness of breath, no. (%)	4 (14)	0 (0)	4 (50)	2 (67)	**0.01**
General weakness, no. (%)	11 (39)	3 (37)	3 (37)	2 (67)	0.88
Malaise, no. (%)	3 (10)	2 (25)	2 (28.5)	1 (33)	0.24
Sore throat, no. (%)	2 (7)	0 (0)	0 (0)	0 (0)	0.99
Rhinorrhea, no. (%)	27 (96)	8 (100)	8 (100)	3 (100)	0.99
Vomiting, no. (%)	10 (36)	2 (25)	0 (0)	3 (100)	**0.01**
Headache, no. (%)	4 (14)	0 (0)	0 (0)	0 (0)	0.57
Chest pain, no. (%)	1 (3.4)	0 (0)	2 (28.5)	1 (33)	**0.05**
Abdominal pain, no. (%)	7 (25)	1(12)	0 (0)	1 (33)	0.37
Joints pain, no. (%)	2 (7)	0 (0)	1 (12)	0 (0)	0.41
Pharynx excaudate, no. (%)	29 (100)	8 (100)	7 (100)	3 (100)	0.99
Conjunctival redness, no. (%)	0 (0)	0 (0)	0 (0)	1 (33)	0.06
Convulsions, no. (%)	3 (11)	2 (25)	0 (0)	0 (0)	0.58
Dispense/Tachypnea					
Abnormal chest radiograph, no. (%)	4 (14)	2 (25)	4 (50)	2 (67)	**0.04**
Respiratory distress, no. (%)	0 (0)	0 (0)	0 (0)	3 (100)	‐

*Note*: Bold values are statistically significant.

Most children were affected by mild and moderate illness, 59.5% and 17%, respectively. 17% were severe and 6.5% were critically ill. There were no statistically significant differences regarding gender in different patients' group (*p* ≥ 0.05), while the presence of the underlying disease had a significant effect on the severity of clinical symptoms in different patient groups (*p* ≤ 0.05).

Among all of the 47 patients recorded, 2 (4%) had SARS‐CoV‐2 RNAemia detected with the aid of rRT‐PCR in serum samples. It is notable that both of these patients were female, one 8‐year‐old and one 15‐year‐old, both of whom had suffering from heart disease and affected by a severe form of the COVID‐19 disease (Table 3).

The occurrence of clinical signs such as fever, sore throat, cough, tachypnea, chest pain, rhinorrhea, diarrhea, abdominal pain, headache, and vomiting were recorded in patients with different severity of symptoms. COVID‐19‐associated symptoms which were statistically different in clinically different patient groups were shortness of breath, vomiting, chest pain, and abnormality in chest (Table 1).

immunological factors for various patient groups are shown in Table 2. Mean white blood cell count increased with increasing severity, but the difference was not significant (*p* ≥ 0.05). The decrease change in mean lymphocyte count from mild to severe group was not statistically significant (*p* ≥ 0.05). Mean platelet and neutrophil cell count changes in different patient groups were not significant in this study, either (*p* ≥ 0.05). A significant difference in the ESR was observed in the group of affected patients in contrast to the previous parameters. d‐dimer and ferritin were only tested in four (three critical patients and one server) and three critical patients, respectively. However, the difference in d‐dimer levels was not statistically significant. C‐reactive protein (CRP) is another important factor which did not show a notable difference among patient groups. Lactate dehydrogenase, troponin, procalcitonin, and hemoglobin were other factors tested in blood samples, and their changes in different groups of hospitalized children were not significant (*p* ≥ 0.05).

**Table 2:** Laboratory data for different groups of pediatric patients with different severity (*N* = 47).

Parameter	Mild ill (*N* = 29)	Moderate ill (*N* = 8)	Severe ill (*N* = 7)	Critically ill (*N* = 3)	*p*‐Value
White blood cell count (×10⁹ cells per L) (mean ± SD)	6.3 (±4.4)	8.9 (±6.3)	12.5 (±6.2)	9.0 (±3.1)	0.23
Lymphocyte count (×10⁹ cells per L) (mean ± SD)	31.2 (±5.2)	23.1 (±7.5)	30.4 (10.3)	11.1 (±0.85)	0.19
Platelet count (×10⁹ cells per L) (mean ± SD)	221 (±74)	237 (±171)	282 (±104)	267.66 (±89)	0.54
Erythrocyte sedimentation rate (mm/h) (mean ± SD)	34.46 (±37.6)	68.62 (±39.46)	13.75 (±22.86)	6.50 (±6.36)	**0.05**
d‐Dimer (mean ± SD)^a^	‐	‐	1914.0	819.6 (±918.60)	0.18
Neutrophil count (×10⁹ cells per L (mean ± SD)	59 (±7.6)	62 (±8.5)	64 (±9.2)	82 (±2.3)	0.38
C‐reactive protein (mg/L) (mean ± SD)	27 (±11.4)	64.6 (±20.5)	39.6 (±12.1)	68.3 (±33.4)	0.23
Lactate dehydrogenase (U/L) (mean ± SD)	692.1 (±137.4)	1165.60 (366.8)	1431.66 (±1045.51)	1470.00	0.33
Ferritin (mean ± SD)^b^	‐	‐	‐	826.30 (±1029)	‐
Troponin (mean ± SD)	24.95 (±20.7)	74.8 (±65.0)	65.8 (±33.9)	29.1 (±21.8)	
Procalcitonin (ng/mL) (mean ± SD)	0.85 (±0.75)	‐	‐	0.51 (±0.29)	0.63
Hemoglobin (g/dL) (mean ± SD)	12.43 (±1.90)	11.32 (±3.60)	12.63 (±2.27)	12.80 (±3.36)	0.65

*Note*: *p*‐value for difference between the four groups were obtained by Kruskal–Wallis test and one‐way ANOVA for nonparametric data and parametric data, respectively. *p*‐value ≤ 0.05 was significant. Bold values are statistically significant.

^a^ D‐Dimer was tested in one severe and three critical patients.

^b^ Ferritin was checked just in two critical patients.

Out of 47 patients, 28 (57%) patients had an underlying disease while both of viremic patients (100%) had an underlying disease. It is worth noting that 78% and 52% of the patients received various types of antibiotics and remdesivir as their primary antiviral COVID‐19 therapy. In 19 of the 47 patients, eight children acquired SARS‐CoV‐2 in the hospital, and data analysis showed that the longer the length of hospitalization, the higher the rate of this respiratory infection.

As shown in Table 3, death was observed in two infants with sever underlying disease. Both cases were hospitalized in the ICUs. The first was an infant with Niemann‐Pick who had frequent seizures and the second case was a diabetic patient.

**Table 3:** Pediatric COVID‐19 infected patients with different types of underlying medical condition (*N* = 28).

	Mild ill (*N* = 14)	Moderate ill (*N* = 8)	Severe ill (*N* = 5)	Critically ill (*N* = 1)	Total (*N* = 28)
Heart diseases	‐	‐	2^a^		2
Nervous diseases	‐	‐	1^b^	1	2
Diabetes	1	‐	1^b^	‐	‐
Liver diseases	1	1	‐	‐	2
Malignancy	1	‐	‐	‐	1
Car accident	‐	2	‐	‐	3
Hemophilia	‐	1	‐	‐	1
G6PD dificiency	1	‐	‐	‐	1
Others	9	4	2	‐	14

^a^ Dead patients (*N* = 2).

^b^ Positive rRT‐PCR blood test (*N* = 2).

## 4 DISCUSSION

This is a prospective study of clinical characteristics in pediatric patients and their correlation with clinical severity, viremia, and immunological factors in Iran.

In this study, 47 individuals with SARS‐CoV‐2 RNA‐positive nasopharyngeal swabs were screened for evidence of RNAemia in paired plasma samples. These results indicate that RNAemia was present in 4% of the samples tested. Various studies in serum, plasma, and whole blood samples revealed varying rates of RNAemia (0%–17%), (0%–20%), and (8%–40%), respectively.^8^ However, most of these data were for adults. In another study conducted on COVID‐19 patients in Iran, 6% of hospitalized children developed viremic illness that was comparable to our study.^16^ Mertz et al. showed that 27% of pediatric COVID‐19 patients had RNemia.^17^ In almost all relevant studies, serum/plasma RNAemia was associated with worse clinical outcomes, the need for supplemental oxygen, the need for PICU care, and longer hospital stay such in our study.

Since the current study had a relatively low number of severe COVID‐19 cases and the patients were sampled only at baseline at enrollment, the number of viremia cases was low.

COVID‐19 symptoms can range from mild fever to ARDS, making diagnosis, prognosis, and further surveillance difficult. In the current study, some series of points played a role in determining the severity of COVID‐19 disease. The most important of these were ventilator use, oxygen with reservoir, vasoactive drug use, and IVIG or corticosteroid therapy.^18^ In our study, 76.5% of children experienced mild and moderate COVID‐19 (59.5% and 17%, respectively), while 23.5% experienced severe and critical forms of the disease (17% and 6.5%). Our data were consistent with those of other studies.^19^, ^20^

The results showed that the most common clinical symptoms of COVID‐19 in children were fever, cough, general weakness, diarrhea, abdominal pain, and malaise, which ranged from 17% to 57%, respectively and they were consistent with those of other studies.^21^, ^22^ Clinical symptoms that differ statistically between patient groups include shortness of breath, vomiting, and chest pain.

ESR was the only abnormal laboratory test result in our study that was different in several patient groups. In Kiani et al. the major important abnormal laboratory parameters in COVID‐19 affected children were CRP, hemoglobin, liver enzymes, and ESR.^23^

In contrast to many studies in adults,^8^, ^9^ this research did not find a significant association between disease severity and viremia. However, the data are consistent with statistics from other studies on children.^16^

The most important reason for the differences in these cases is probably the change of viral strains. This study was conducted during the outbreak of the Delta virus strain, while previous studies had occurred with other strains, such as Alpha and Gamma. Another reason might be differences in the genetics of different population groups.

Although this study uses a highly sensitive Taq Man real‐time PCR method with two pairs of primers and probes, different sensitivities are effective in determining the percentage of viremia in hospitalized patients.

Fortunately, in the current study, a 60‐day mortality rate was not significant even in the presence of different complications in a high percentage of patients. However, the current study reports only two deaths (4%) in infants with a complicated underlying medical condition. A study by Pourakbari and colleagues found a pediatric mortality rate of 10%.^24^

In the current study, as with others, an underlying medical condition appears to be a key factor in determining COVID‐19 disease in children, but there is a lack of evidence to judge the importance and strength of our findings; therefore, it suggests more research.^25^

The small sample size of our study is a main limitation, especially the sample size of patients with SARS‐CoV‐2 viremia, which may affect the statistical results.

## 5 CONCLUSION

Our data confirmed that the severity of COVID‐19 varied among children infected with SARS‐CoV‐2 and was low, as in other studies. Dyspnea, vomiting, chest pain, and chest abnormalities were critical elements that occurred significantly more frequently in critically ill patients. Our results suggest that ESR is the only factor that distinguishes COVID‐19 disease severity among immunological factors and, in contrast to many studies in adult, no significant association was observed between viremia and disease severity in children.

## AUTHOR CONTRIBUTIONS

**Marzieh Jamalidoust**: Conceptualization; project administration; software; supervision; visualization; writing—original draft; writing—review and editing. **Seyedeh Sedigheh Hamzavi**: Conceptualization; funding acquisition. **Eslam Shorafa**: Conceptualization; investigation; supervision. **Mandana Namayandeh**: Data curation; methodology. **Laiba Batool**: Data curation; visualization. **Seyedeh Narges Abootalebi**: Data curation; investigation.

## CONFLICT OF INTEREST STATEMENT

The authors declare no conflict of interest

## ETHICS STATEMENT

The project has been approved by Shiraz University of Medical Sciences (Ethics code Number: IR.SUMS.REC.1400.691). All authors acquiesced to the final version of the manuscript.

## TRANSPARENCY STATEMENT

The lead author Marzieh Jamalidoust, Seyedeh Sedigheh Hamzavi affirms that this manuscript is an honest, accurate, and transparent account of the study being reported; that no important aspects of the study have been omitted; and that any discrepancies from the study as planned (and, if relevant, registered) have been explained.

## Data Availability

The data that support the findings of this study are available from the corresponding author upon reasonable request.
